# Metabolomics to Assess Response to Immune Checkpoint Inhibitors in Patients with Non-Small-Cell Lung Cancer

**DOI:** 10.3390/cancers12123574

**Published:** 2020-11-30

**Authors:** Veronica Ghini, Letizia Laera, Beatrice Fantechi, Francesca del Monte, Matteo Benelli, Amelia McCartney, Leonardo Tenori, Claudio Luchinat, Daniele Pozzessere

**Affiliations:** 1 Cirmmp, Via Luigi Sacconi 6, 50019 Sesto Fiorentino, Florence, Italy; ghini@cerm.unifi.it; 2 Magnetic Resonance Center, CERM, University of Florence, Via Luigi Sacconi 6, 50019 Sesto Fiorentino, Florence, Italy; tenori@cerm.unifi.it; 3 Sandro Pitigliani, Department of Medical Oncology, Hospital of Prato, via Suor Niccolina Infermiera, 20/22, 59100 Prato, Italy; l.laera@miulli.it (L.L.); beatrice.fantechi@uslcentro.toscana.it (B.F.); francesca.delmonte@uslcentro.toscana.it (F.d.M.); amelia.mccartney@uslcentro.toscana.it (A.M.); 4 Department of Oncology, Miulli hospital, Acquaviva delle Fonti, 70021 Bari, Italy; 5 Bioinformatics Unit, Hospital of Prato, via Suor Niccolina Infermiera, 20/22, 59100 Prato, Italy; matteo.benelli@uslcentro.toscana.it; 6 Department of Chemistry, University of Florence, via della Lastruccia 3, 50019 Sesto Fiorentino, Florence, Italy

**Keywords:** immune checkpoint inhibitors, non-small cell lung cancer, metabolomics, nuclear magnetic resonance

## Abstract

**Simple Summary:**

Recently, immunotherapy has presented new opportunities for clinical development in the treatment of non-small cell lung cancer (NSCLC). Although effective in sustaining overall survival in several clinical trials, not all the NSCLC patients respond to these treatments. Thus, a better patient selection, as well as the identification of predictive biomarkers of treatment efficacy, are of paramount importance. In this work, metabolomics was used with the aim of identifying responder with respect to non-responder subjects. We show that the metabolomic fingerprint of serum samples, collected before therapy, acts as a predictive biomarker to treatment response. Prospective identification of subjects that will benefit from immunotherapy could improve patient stratification, thus optimizing the treatment and avoiding unsuccessful strategies.

**Abstract:**

In the treatment of advanced non-small cell lung cancer (NSCLC), immune checkpoint inhibitors have shown remarkable results. However, not all patients with NSCLC respond to this drug treatment or receive durable benefits. Thus, patient stratification and selection, as well as the identification of predictive biomarkers, represent pivotal aspects to address. In this framework, metabolomics can be used to support the discrimination between responders and non-responders. Here, metabolomics was used to analyze the sera samples from 50 patients with NSCL treated with immune checkpoint inhibitors. All the samples were collected before the beginning of the treatment and were analyzed by NMR spectroscopy and multivariate statistical analyses. Significantly, we show that the metabolomic fingerprint of serum acts as a predictive “collective” biomarker to immune checkpoint inhibitors response, being able to predict individual therapy outcome with > 80% accuracy. Metabolomics represents a potential strategy for the real-time selection and monitoring of patients treated with immunotherapy. The prospective identification of responders and non-responders could improve NSCLC treatment and patient stratification, thus avoiding ineffective therapeutic strategies.

## 1. Introduction

Lung cancer (LC) is the leading cause of cancer-related mortality worldwide. Eighty-five percent of patients with LC are affected by non-small cell lung cancer (NSCLC), with the majority of patients presenting with advanced, unresectable disease at the time of diagnosis [1]. Current treatment strategies for advanced NSCLC include chemotherapy, targeted agents, immunotherapy, or chemo-immunotherapy. Treatment choices are made according to tumor histology as well as PDL-1 TPS (tumor proportion score) and the presence of specific genomic alterations. However, the majority of patients does not attain durable disease control, and the five-year survival rates remain very low. Thus, more effective strategies are needed.

The ability of LC to evade the body’s immune surveillance by utilizing certain “immune checkpoints” that normally protect against autoimmunity and inflammation has been well characterized [2]. Immune checkpoint inhibitors (ICIs) have become an attractive treatment modality for chemo-refractory solid neoplasms. These agents reactivate T lymphocyte-mediated immune response against the tumor in the microenvironment by blocking immune checkpoint molecules, including cytotoxic T-lymphocyte antigen-4 (CTLA-4), programmed cell death protein 1 (PD-1), and its ligand (PDL-1) [3]. Antibody-directed therapies against these checkpoints have shown remarkable early success in many malignancies, and play a critical role in the management of advanced lung cancer and other tumour types [4].

Several monoclonal antibodies directed to the PD-1 receptor or its ligand PDL-1 (for example nivolumab, pembrolizumab, and atezolizumab) are already established in daily practice, with others in preclinical development. Clinical trials with these agents have shown rapid and durable responses in about 14–20% of pre-treated patients with advanced NSCLC [5,6,7,8]. Importantly, although progression-free survival data are modest (20% progression-free survival at one year), reported sustained survival outcomes are remarkable. In the 27-month follow-up of a cohort of 129 patients with NSCLC treated with nivolumab, two-year survival was 24% in the overall population and 42% in the subset of patients treated at the dose selected for further development (3 mg/kg every 2 weeks); three-year survival was 18% in the overall population and 27% in the development dose subset of patients [9]. Based on these data, immunotherapy has established an important role in the first line of treatment of advanced disease as a monotherapy or in combination with chemotherapy [10], as well as in limited-stage disease [11].

However, not all patients respond to ICIs, with modest response rates (approximately 20% or less in LC) and associated high treatment costs. Thus, there is crucial interest in the discovery and development of biomarkers that can predict which patients are most likely to respond and benefit from ICIs, thus improving clinical decision-making and disease management [12]. 

Clinical efficacy of ICIs appears independent of histology, but in most trials, a greater benefit was observed in smokers and in patients with positive PDL-1 expression. PDL-1 expression has proved to be an effective patient selector but is far from being a true predictive marker. Patients with PDL-1-positive NSCLC do not derive universal benefit from these agents, while some tumors with low or negative expression of PDL-1 do show response [4]. Additionally, the utilization of tumor mutational burden (TMB; the overall quantity of aberrant nucleotide sequences a given tumor may harbor) has produced conflicting results, casting doubt on its ability to be a predictor of response to immunotherapy [13].

Routine clinical practice can be complicated by the fact that radiological indications of tumor response and progression associated with immune checkpoint blockade therapy can be different from those observed in patients receiving a conventional chemotherapeutic and/or molecular-targeted agents. Pseudo-progression can manifest itself as an increase in the size of target lesions, or even as the development of new lesions on imaging. However, such paradoxical changes are not necessarily due to resistance to treatment, but rather a sign of treatment effect [14]. 

Metabolomics deals with the characterization of the metabolome, i.e., the ensemble of metabolites presents in a cell, a tissue, a biofluid or in an entire organism [15]. The human metabolome is composed by several thousands of small molecules produced by the organism and by its microflora, as well as deriving from environmental factors [16]. It is a dynamic entity that may change as a result of lifestyle, stress and, most importantly, onset of pathologies and medical treatment [17,18,19,20,21,22]. In the field of oncology, metabolomics has shown potential for early diagnosis, prognosis, individual monitoring and drug therapy design [23,24,25]. Nuclear magnetic resonance (NMR)-based metabolomics is an efficient and highly reproducible platform for the analysis of biofluids, such as blood serum or plasma [26,27]. The use of biofluids for screening and monitoring has the advantage of being minimally invasive, can be applied on a large scale, and may provide significant information on tumor -specific phenotype [26,27]. In this framework, NMR metabolomic fingerprinting of biofluids has been increasingly used to establish a metabolomic signature both before and after a given drug therapy that might inform on treatment outcomes [28,29,30]. 

In this study, we conducted an NMR-based metabolomic investigation of sera samples from 50 patients treated with immune checkpoint inhibitors for NSCLC. All the analyzed samples were collected before the beginning of the treatment with the aim to find predictive metabolomic profiles of the response to immune checkpoint inhibitors.

## 2. Results

Serum sample from 53 patients with advanced NSCLC were analyzed. Patient and tumor characteristics and type of treatment are reported in Table S1. Thirty-four patients were treated with nivolumab as second line therapy after chemotherapy, and 19 patients were treated with pembrolizumab as first-line therapy. Overall, 19 patients presented with a squamous cell carcinoma (SqCC), 31 with adenocarcinoma (non-SqCC), and three with NOS (not otherwise specified) carcinoma. 

Due to the higher number of samples collected, our analyses mainly focused on nivolumab-treated subjects.

### 2.1. NSCLC Nivolumab-Treated Cohort

**Figure 1:**
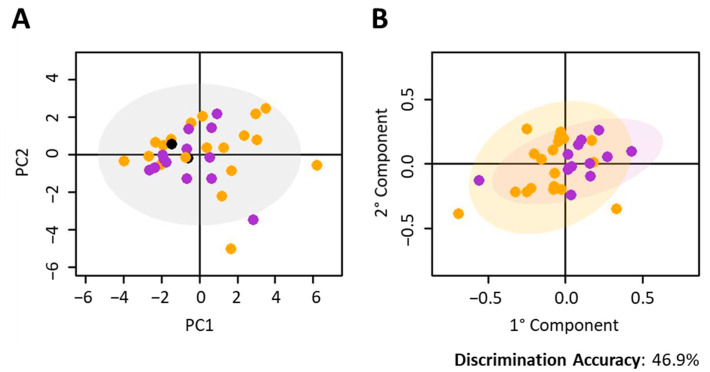
Nivolumab-treated cohort. Discrimination between the metabolomic profiles of baseline serum samples (T0) from different histology of NSCLC. Score plots of (**A**) PCA analysis of all samples; (**B**) O-PLS analysis of samples with squamous carcinoma and adenocarcinoma. In each score plot, each dot represents a different serum sample. Orange dots: adenocarcinoma; green dots: squamous cell carcinoma; black dots: NOS histology.

Firstly, we investigated if the metabolomic profiles of baseline T0 serum samples from nivolumab-treated patients were significantly different according to histological profile (Figure 1). Applying principal component analysis (PCA) and orthogonal partial least squares (O-PLS) models, we compared the sera from patients with squamous cell (*N* = 19), adenocarcinoma (*N* = 13) and NOS carcinoma (*N* = 2). The T0 metabolomic phenotypes of the patients with different histology types were not significantly different (2-group discrimination accuracy of 48.0% (*p*-value: 0.86), squamous vs. adenocarcinoma, DQ^2^ of −0.02) (Figure 1).

As there were no significant differences according to histological subtype, all the tumor types were analysed together from here on. 

Multivariate statistical analysis was then used to analyse T0 samples to extract a serum metabolomic fingerprint able to discriminate between responder and non-responder subjects, with the final aim of obtaining a priori information about treatment outcome.

The first attempt to establish a classification between responder and non-responder subjects was performed according to the first radiological assessment (Table 1). In this framework, iRECIST criteria were used. “Responders” were defined as those subjects who achieved complete response (iCR), partial response (iPR), or stable disease (iSD), whereas “non-responders” were those with unconfirmed progressive disease (iUPD), confirmed progressive disease (iCPD) or hyper-progression (iHP). Subjects who died before the first radiological assessment were classified as iHP.

**Figure 2:**
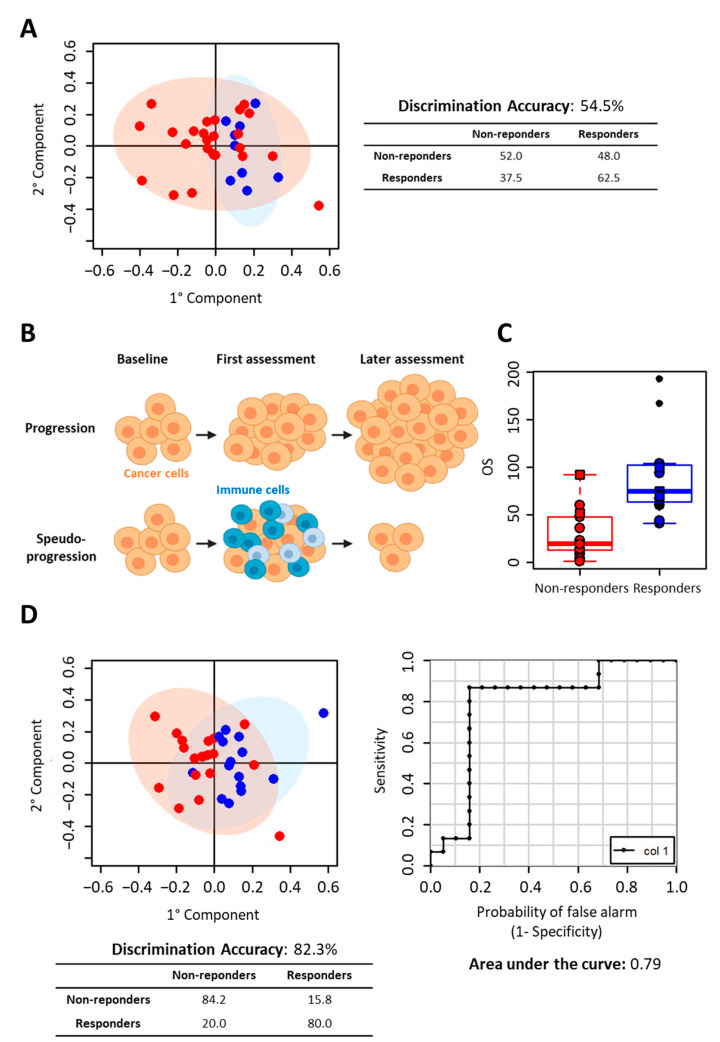
Prediction of Nivolumab response. (**A**) Discrimination between responders and non-responders according to the first radiological assessment. O-PLS analysis of T0 samples. Score plot, PC1 vs. PC2, and corresponding confusion matrix. Red dots: non-responder subjects; blues dots: responder subjects. (**B**) Scheme of disease progression vs. pseudo-progression in the presence of immune cells attacking the tumour. (**C**) Boxplots of OS values of the subject. Red dots: non-responder subjects; blue dots: responder subjects (subjects still on alive are marked with black dots). (**D**) Discrimination between responders and non-responders according to the second radiological assessment. O-PLS analysis of T0 serum samples. Score plot, PC1 vs. PC2, and corresponding confusion matrix (left panel); ROC curve derived from O-PLS cross-validation (right panel). Red dots: non-responder subjects; blues dots: responder subjects.

O-PLS analysis was not able to discriminate partial/complete responders (9 subjects) from non-responders (25 subjects), obtaining a discrimination accuracy lower than 60% (*p*-value: 0.76) and a DQ^2^ of −0.30 (Figure 2A). In the case of immunotherapy, the therapy response estimation through radiological assessment may not be easy to evaluate, due to characteristic patterns of response to treatment, including the phenomenon of the pseudo-progression. Pseudo-progression does not reflect tumour cell growth but may be misclassified as disease progression (Figure 2B).

To overcome the difficulties of evaluating treatment responses to include clinical judgment, we decided to take into account the second radiological assessment (Table 1). In daily clinical practice, if a minimum or asymptomatic progression of disease is observed, treatment is continued until the patient becomes symptomatic, as suggested by expert consensus [1].

All subjects who discontinued the treatment at the second radiological assessment, or before, due to confirmed progressive disease, hyper-progression, or death, were considered as non-responders. Conversely, all subjects continuing therapy until the third or further radiological assessments were considered as responders. Thus, 15 subjects were considered as responders, and 19 subjects as non-responders. As expected, the time to treatment failure (TTF) values of responder and non-responder subjects (Table 1) are significantly different (median TTF in non-responders: 10 weeks versus median TTF in responders: 54 weeks; *p*-value < 0.001). Moreover, looking at overall survival (OS), the OS median values of responder and non-responder subjects are significantly different, Table 1 and Figure 2C (median OS in non-responders: 19.5 weeks versus median OS in responders: 75 weeks; *p*-value < 0.001).

Using this criterion, the discriminatory power of the O-PLS analysis significantly improved, the model being able to discriminate between responders and non-responders with an accuracy of 82% (DQ^2^: 0.16); accordingly, the ROC curve derived from O-PLS cross-validation showed an area under the curve (AUC) of 0.79. In this model only six out of 33 subjects were misclassified (Figure 2D). The O-PLS predicting power was assessed for significance against the null hypothesis of no prediction accuracy in the data, by means of 500 randomized class permutation tests. The average discrimination accuracy obtained after randomization was 58.3%, demonstrating the significance of our results (*p*-value: 0.006).

No apparent common characteristics are shared among the six misclassified subjects (MetL13, MetL19, MetL41, MetL45, MetL50, and MetL57): four were male (two had squamous carcinoma and two adenocarcinoma), and two were female—one with squamous carcinoma and the other with adenocarcinoma.

Due to the relatively small number of samples, these results were obtained using a leave-one-out cross-validation scheme. By using a Monte Carlo cross-validation scheme, the discrimination accuracy remained very high, at 78% (*p*-value of 0.006), with a DQ^2^ of 0.17. 

### 2.2. NSCLC Pembrolizumab-Treated Cohort

The serum samples of 19 patients treated with pembrolizumab as first line therapy were also collected (Table S1). Due to the small sample number size, these cannot be used to build a statistically significant model aimed at the identification of responders to pembrolizumab. Thus, to explore the possibility that a metabolomic signature able to discriminate between responders and non-responders to anti PD-1 therapy can be established in the context of pembrolizumab therapy, we used the discriminatory power of the O-PLS model created in nivolumab-treated subjects to predict the outcome of pembrolizumab-treated patients (Table 2).

**Figure 3:**
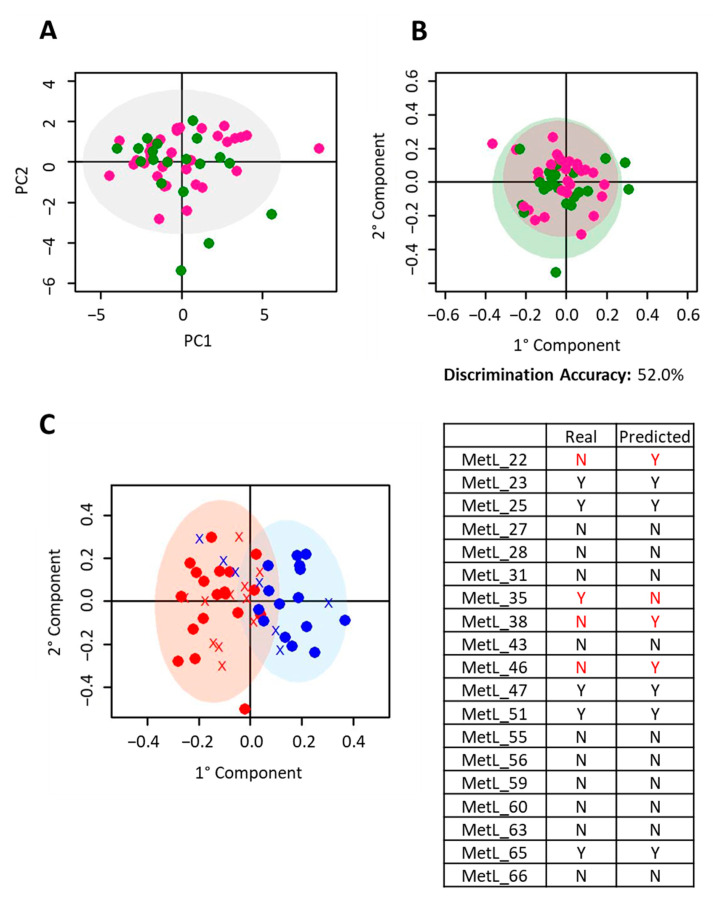
Prediction of Pembrolizumab subjects. Discrimination between the metabolomic profiles of baseline serum samples (T0) from nivolumab- and pembrolizumab- treated subjects. Score plots of (**A**) PCA analysis; (**B**) O-PLS analysis. In each score plot, each dot represents a different serum sample. Purple dots: nivolumab; green dots: pembrolizumab. (**C**) Prediction of pembrolizumab-treated subjects. O-PLS analysis of T0 serum samples. Score plot, PC1 vs. PC2, and corresponding confusion matrix. Red dots: nivolumab responder subjects; blues dots: nivolumab non-responder subjects. Red crosses: pembrolizumab subjects predicted as non-responders; blue crosses: pembrolizumab subjects predicted as responders.

Firstly, we investigated if the metabolomic profiles of T0 samples from nivolumab- and pembrolizumab-treated patients were significantly different. Applying PCA and O-PLS models, we detected that the serum metabolomic phenotypes were not significantly different according to treatment group, with a 2-group discrimination accuracy of 52.0% (*p*-value: 0.84) with a DQ^2^ of −0.43 (Figure 3A,B). Then, the O-PLS model created on nivolumab-treated patients was used to predict the pembrolizumab-treated subjects. Fifteen out of the nineteen pembrolizumab-treated subjects were correctly classified, obtaining a total prediction accuracy of 79% (Figure 3C). These results indicated that a serum fingerprint able to discriminate responders exists both for nivolumab and pembrolizumab treatment.

### 2.3. Metabolite Analysis

**Figure 4:**
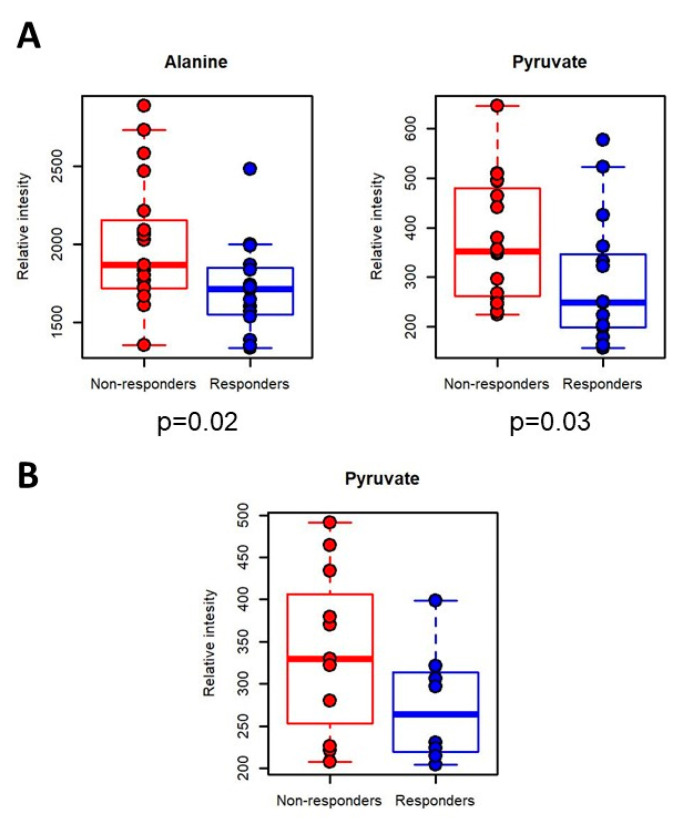
Metabolites analysis. Box plot of the serum levels of the significantly different metabolites for the comparison between responders and non-responders in (**A**) nivolumab-treated patients; (**B**) pembrolizumab-treated patients.

NMR spectra were analyzed to identify which metabolites were the main contributers to the discrimination between responders and non-responders (Table S2 and Figure S1). Considering nivolumab-treated patients, non-responder and responder subjects were characterized by significantly (*p*-value < 0.05, FDR < 0.05) different levels of the amino acid alanine and of pyruvate. In particular, responders are characterized by lower serum levels of the two above mentioned metabolites (Figure 4A). Interestingly, the same behavior of pyruvate is confirmed in pembrolizumab-treated patients. Pyruvate serum levels, even not significantly, are lower in non-responders with respect to responders (Figure 4B).

## 3. Discussion

Cancer immunotherapy has changed conventional treatment paradigms by expanding treatment options for patients with NSCLC. However, despite current success, the response rate to ICIs in advanced NSCLC is around 30%. Thus, there is a growing need to identify predictive and prognostic biomarkers to guide patient selection. The basic principles underlying a good biomarker include analytical validity (reliability and reproducibility), as well as clinical utility. Patient selection based on the expression of PDL-1, as detected by immunohistochemistry and the tumour mutational burden, is used to augment prediction of the likelihood of response to immunotherapy [31,32,33]. In recent years, efforts have been made to identify more reliable methods for the prediction of the response to immune checkpoint inhibitors with controversial results [34,35]. The molecular basis of tumor immunogenicity and cancer immune-escape involves complex mechanisms, and are not yet well understood. The extreme dynamism of the immune system likely explains in part the difficulties encountered in defining a true predictive marker. The study of cellular metabolism performed at the beginning of treatment may overcome the obstacles described above.

Here, we explored the role of metabolomic fingerprinting of serum samples as a predictive biomarker of response to immunotherapy, and we identified a serum metabolomic “signature” that differentiates responder and non-responder patients with NSCLC to nivolumab and pembrolizumab. To our knowledge, this represents the first study that applied NMR-based metabolomics of serum to predict responsiveness to anti PD-1 therapy in NSCLC. A mass spectrometry (MS)-based metabolomic analysis in plasma samples from 55 patients with NSCLC treated with nivolumab has been recently performed [36]. In that paper Hatae et al. demonstrated that a combination of 4 plasma metabolites and several T cell markers could serve as good biomarkers for responder identification (AUC = 0.96). The four metabolite markers include molecules related to gut microbiota (hippuric acid), fatty acid oxidation (butyryl-carnitine), and two redox-related metabolites (cystine and glutathione disulfide) [36]. Using the same methodological approach, in another recent investigation, metabolomic profiling of gut microbiota of a small cohort of patients with NSCLC treated with nivolumab were analysed [37]. In this case, two metabolites—2-pentanone (ketone) and tridecane (alkane)—were significantly associated with early progression. Conversely, short chain fatty acids propionate and butyrate, as well as lysine and nicotinic acid were significantly associated with long-term beneficial effects [37]. Moreover, MS-based metabolomic analysis in serum samples has also recently been used as a tool to investigate metabolic alterations in response to immune checkpoint blockade in advanced melanoma and renal cell carcinoma patients treated with nivolumab (17). In this setting, an increase in serum kynurenine/tryptophan ratio was identified as an adaptive resistance mechanism associated with worse overall survival [38]. All these studies underline the multifactorial and complex nature of immune systems and immunotherapy efficacy, highlighting individual gut microflora as one of the important players. Some evidence has demonstrated that the tumor microenvironment may also play and important role in inhibiting T cell functionality and immunotherapy efficacy [39]. The tumor microenvironment, in fact, can be metabolically hostile due to insufficient vascular exchange and cancer cell metabolism. Cancer cells rapidly consume nutrients and produce lactic acid, leading to hypoxia and high acidity [40]. In this framework, several studies have shown the association of hypoxia with a more aggressive cancer phenotype [41,42]. In particular, lack of oxygen stabilizes HIF-1α, which increases glycolysis and decreases oxidative phosphorylation [41,42]. Interestingly, in the serum samples of non-responder subjects we detected significantly higher levels of pyruvate and alanine along with, even not statistically significant, higher lactate and glycine levels, and lower citrate levels. Higher levels of pyruvate, ala, gly and lactate are all indicative of increased glycolysis and lower levels of citrate are indicative of decreased TCA pathway [43,44]. Thus, we can hypothesize that, in non-responder patients, the tumor microenvironment is particularly hostile and T-cells are not able to overcome these metabolic challenges. It is important to underline that the significance of the univariate analysis performed on single metabolite levels strongly relies on the number of subjects included in the study. Thus, further investigations to confirm this hypothesis are needed.

The strength of our work relies on the fact that, although the sensitivity of NMR is low compared to MS, the ^1^H NMR fingerprint approach take advantage of the its intrinsically untargeted nature and high reproducibility that allowed us to identify metabolomic signatures that are independent from metabolite assignment and act as stronger biomarkers with respect to a single molecules or a panel of few molecules [27].

Our study is strengthened by the use of the sera-derived metabolomic fingerprints of patients treated with pembrolizumab as a small independent validation group, which confirmed the results obtained in nivolumab-treated patients. We chose to combine patients receiving nivolumab and pembrolizumab because both treatments have the same mechanism of action, since they both interfere with the interaction between PD-1 and PDL-1, an interaction that downregulates T cells, allowing cancer cells to evade immune surveillance [45].

## 4. Materials and Methods 

### 4.1. Patient Recruitment and Serum Sample Collection

Patients with advanced NSCLC, older than 18 years, were enrolled at the Hospital of Prato, Prato, Italy (Table S1). We collected blood samples from all patients just prior to the initiation of the first administration of an immune checkpoint inhibitor treatment according to normal first and second or third line clinical practice [5,6,33]. All patients gave their informed consent for inclusion before they participated in the study. The study was conducted in accordance with the Declaration of Helsinki.

This research is approved by Comitato Etico Regionale per la Sperimentazione Clinica della Toscana - sezione AREA VASTA CENTRO on 28 March 2017, the ethic code is “Rif CEAVC—2018-438 (ID 10812)”.

The enrolled patients are consecutive and received pembrolizumab alone as first line treatment [33] if their tumors had a PDL1 expression greater than or equal to 50% or nivolumab as second- or third-line treatment [5,6] regardless of the PDL1 expression value after platinum failure. 

Nivolumab (240 mg total dose) was administered as 30 min infusion every 2 weeks and continued until disease progression, unacceptable toxicity, withdrawal of consent, or loss to follow up. Pembrolizumab (200 mg total dose) was administered every 3 weeks and continued until disease progression, unacceptable toxicity, withdrawal of consent, or lost to follow up.

During treatment, each subject was monitored, and efficacy assessment was periodically evaluated through radiological evaluations (and according to iRECIST [46] criteria) every 8 or 9 weeks, for nivolumab and pembrolizumab, respectively. Each patient was classified as complete/partial responder or non-responder.

Overall survival (OS), calculated as the time from randomization to death, and time to treatment failure (TTF) [47], calculated as the time from randomization to treatment discontinuation for any reason, including disease progression, treatment toxicity, patient preference, or death [48], were also calculated for each patient.

Blood samples were collected from each patient before starting nivolumab or pembrolizumab treatment (T0 samples). Serum samples were prepared, collected, and stored following standard operating procedures guaranteeing high quality biological samples for metabolomic analysis [49,50]. Briefly, blood samples were collected from after a minimum of 8 h fasted patients and processed within two hours of blood collection. The blood was allowed to clot in an upright position for 30−60 min at room temperature (RT) before centrifugation (1500 RCF for 10 min at RT). Serum samples were immediately stored at −80 °C.

### 4.2. NMR Analysis 

NMR samples were prepared according to standard procedures [50]. Frozen serum samples were thawed at room temperature and shaken before use.

A total of 350 μL of sodium phosphate buffer (70 mM Na_2_HPO_4_; 20% (*v/v*) ^2^H_2_O; 6.1 mM NaN_3_, 4.6 mM sodium trimethylsilyl [2,2,3,3−^2^H_4_] propionate (TMSP), pH 7.4) was added to 350 μL of each serum sample, and the mixture was homogenized by vortexing for 30 s. A total of 600 μL of each mixture was transferred into a 5.00 mm NMR tube (Bruker BioSpin) for the analysis.

^1^H-NMR spectra for all samples were acquired using a Bruker 600 MHz spectrometer (Bruker BioSpin) operating at 600.13 MHz proton Larmor frequency and equipped with a 5 mm PATXI ^1^H−^13^C−^15^N and ^2^H-decoupling probe including a z axis gradient coil, an automatic tuning-matching (ATM) and an automatic and refrigerated sample changer (SampleJet, Bruker BioSpin). A BTO 2000 thermocouple served for temperature stabilization at the level of approximately 0.1 K at the sample. Before measurement, samples were kept for 5 min inside the NMR probe head, for temperature equilibration at 310 K.

For each serum sample, three monodimensional ^1^H NMR spectra were acquired at 600 MHz with water peak suppression and different pulse sequences that allowed the selective observation of different molecular components:

(i) A standard NOESY 1Dpresat (noesygppr1d.comp; Bruker BioSpin) pulse sequence (using 32 scans, 98,304 data points, a spectral width of 18,028 Hz, an acquisition time of 2.7 s, a relaxation delay of 4 s and a mixing time of 0.1 s.) to obtain a spectrum in which signals of both metabolites and high molecular weight molecules (lipids and lipoproteins) are visible.
(ii) A standard CPMG (cpmgpr1d.comp; Bruker BioSpin) pulse sequence (using 32 scans, 73,728 data points, a spectral width of 12,019 Hz and a relaxation delay of 4 s.), designed for the selective observation of small molecule components in solutions containing macromolecules.
(iii) A standard diffusion-edited (ledbgppr2s1d.comp; Bruker BioSpin) pulse sequence (using 32 scans, 98,304 data points, a spectral width of 18,028 Hz and a relaxation delay of 4 s.), for the selective observation of macromolecule components in solutions containing small molecules.

### 4.3. Spectral Processing

Free induction decays were multiplied by an exponential function equivalent to a 0.3 Hz line-broadening factor before applying Fourier transform. Transformed spectra were automatically corrected for phase and baseline distortions and calibrated (glucose doublet at δ 5.24 ppm) using TopSpin 3.5 (Bruker BioSpin). Each spectrum in the region 10.00–0.2 ppm was segmented into 0.02 ppm chemical shift bins, and the corresponding spectral areas were integrated using the AMIX software. Binning is a means to reduce the number of total variables and to compensate for small shifts in the signals, making the fingerprint analyses more robust and reproducible. The area of each bin was normalized to the total spectral area, calculated with exclusion of the water region (4.50–5.00 ppm).

### 4.4. Statistical Analysis

Multivariate analyses were applied on binned spectra using R software using in-house scripts (the scripts could be provided upon reasonable request). 

Principal component analysis (PCA) was used as unsupervised exploratory analysis to obtain a preliminary outlook of the data (presence of clusters or outliers). Orthogonal Partial Least Squares Discriminant Analysis (OPLS-DA) was used to increase supervised data reduction and obtain the best discrimination between the analyzed groups.

The global accuracy for classification was assessed by means of both a Leave-one-out cross-validation scheme and a Monte Carlo cross-validation scheme. Accordingly, each dataset was randomly divided into a training set (90% of the data) and a test set (10% of the data). The training set was used to build the model, whereas the test set was used to validate its discriminant and predictive power; this operation was repeated 500 times. 

For each model, the resultant confusion matrix was reported, and its discrimination accuracy, specificity and sensitivity were estimated according to standard definitions. Each classification model was also validated using a permutation test; the permutation was repeated 500 times and the resulting *p*-value was calculated. Discriminant Q^2^ values (DQ^2^) were calculated according to Westerhuis et al. [51].

The metabolites, whose peaks in the spectra were well resolved, were assigned and their concentration levels analyzed, as shown in Table S2. The assignment was performed using an internal NMR spectral library of pure organic compounds, public databases such as the Human Metabolome Database, stored reference NMR spectra of metabolites, and using literature data. Matching between new NMR data and databases was performed using the Assure NMR software (Bruker, Billerica, MA, USA).

The nonparametric Wilcoxon–Mann–Whitney test was used for the determination of the meaningful metabolites. A *p*-value < 0.05 was considered statistically significant. In order to reduce false discoveries, false discovery rate correction (FDR) was then applied using the Benjamini and Hochberg method.

## 5. Conclusions

In conclusion, we showed that the individual outcome of anti PD-1 therapy in patients with NSCLC can be predicted by metabolomic analysis of serum collected before commencing treatment, with >80% accuracy. Moreover, metabolomic prediction appears to be independent of ICI agent, making metabolomics a potential strategy for the real-time selection and monitoring of patients treated with immunotherapy. This may address the demands of modern personalized medicine, in which treatment decisions are tailored based on a subject’s individual subtype, with the final aim of avoiding ineffective therapy and improving patient care. A larger confirmatory study is ongoing.

## Figures and Tables

**Table 1:** NSCLC Nivolumab-treated cohort. For each patient the (i) number of cycles of nivolumab therapy performed, (ii) time of treatment failure (TTF), (iii) overall survival (OS), (iv) iRECIST classification for the radiological assessments (RA), are reported: complete response (iCR), partial response (iPR), stable disease (iSD), unconfirmed progressive disease (iUPD), confirmed progressive disease (iCPD) and hyper-progression (iHP). Patients continuing the therapy are highlighted in grey.

Subject Number	N° Cycles	TTF (Weeks)	OS (Weeks)	1 RA	2 RA	3 RA	4 RA	5 RA	6 RA	7 RA	8 RA	9 RA	10 RA	11 RA	12 RA
MetL01	4	8	10	na (HP)											
MetL05	24	59	94	iSD/PR	iSD	iUPD	iCPD								
MetL06	4	8	13	iUPD											
MetL07	84	186+	193+	iPR	iPR	iPR	iPR	iPR	iPR	iPR	iPR	iPR	iPR	iPR	iPR
MetL09	19	38	67	iSD	iUPD	iUPD	iCPD								
MetL10	10	18	20	iSD	iSD										
MetL11	9	23	60	iUPD	iCPD										
MetL12	23	45	60	iUPD	iPR	iSD	iPD								
MetL13	29	140+	167+	iUPD	iUPD	iPR	iPR	iUPD	iUPD	iPR	iSD	iSD	iSD		
MetL15	2	2	5	na (HP)											
MetL17	19	41	103	iUPD	iUPD	iCPD									
MetL19	27	66	104	iUPD	iUPD	iSD	iSD	iCPD							
MetL20	4	6	8	na (HP)											
MetL24	6	10	48	iPD											
MetL26	6	10	36	iPD											
MetL29	3	5	60	na (HP)											
MetL30	2	4	14	HP											
MetL32	8	17	19	iUPD											
MetL33	13	35	41	iSD	iSD	iUPD	iCPD								
MetL34	2	2	4	na (HP)											
MetL36	8	16	21	iUPD	iCPD										
MetL37	17	38	44	iUPD	iUPD	iCPD									
MetL39	4	7	14	iUPD											
MetL40	47	99+	102+	iPR	iPR	iPR	iPR	iPR	iPR	iPR					
MetL41	6	12	23	iUPD	iCPD										
MetL42	18	39	100+	iUPD	iUPD	iUPD	iCPD								
MetL44	11	20	92+	iUPD	iCPD										
MetL45	8	15	19	iUPD	iCPD										
MetL48	19	37	75+	iUPD	iPR	iPD									
MetL49	1	1	1	na (HP)											
MetL50	35	70+	73+	iPR	iPR	iPR	iPR								
MetL52	29	58+	70+	iUPD	iUPD	iUPD	iUPD	iUPD	iCPD						
MetL53	19	54+	59+	iPR	iPR	iSD	iPR								
MetL57	7	15	52+	iUPD	iCPD										

**Table 2:** NSCLC Pembrolizumab -treated cohort. For each patient the (i) number of cycles of pembrolizumab therapy performed, (ii) time of treatment failure (TTF), (iii) overall survival (OS), (iv) PD-L1 expression levels; (v) iRECIST classification for the radiological assessments (RA), are reported: complete response (iCR), partial response (iPR), stable disease (iSD), unconfirmed progressive disease (iUPD), confirmed progressive disease (iCPD) and hyper-progression (iHP). Patients considered responders are highlighted in grey.

Subject Number	N° Cycles	TTF (Week)	OS (Week)	PD-L1	1 RA	2 RA	3 RA	4 RA	5 RA	6 RA	7 RA	8 RA	9 RA
MetL22	35	115+	118+	80	iPR	iPR	iPR	iPR	iPR	iSD	iSD	iUPD	iSD
MetL23	36	115+	116+	60	iPR	iPR	iCR	iCR	iCR	iSD	iSD	iSD	
MetL25	33	106+	109+	70	iSD	iSD	iSD	iSD	iSD	iSD	iSD	iSD	iSD
MetL27	3	6	14	80									
MetL28	8	24	39	70	iPR	iUPD	iCPD						
MetL31	3	8	10	70									
MetL35	11	37	39	70	iSD	iUPD	iUPD	iCPD					
MetL38	26	82+	87+	60	iPR	iPR	iSD	iSD	iSD				
MetL43	1	5	5	60									
MetL46	17	62+	66+	60	iUPD	iPR	iPR	iPR	iPR				
MetL47	17	54+	56+	80	iPR	iUPD	iSD						
MetL51	14	45+	49+	70	iPR	iPR	iSD						
MetL55	1	1	1	80									
MetL56	3	9	17	90	iPD								
MetL59	3	6	xx	80	iPD								
MetL60	5	15	19	60	iSD								
MetL63	4	20	23	50	iUPD	iCPD							
MetL65	8	22	30+	90	iPR	iUPD							
MetL66	7		26	90	iUPD	iCPD							

## Supplementary Materials

The following are available online at https://www.mdpi.com/2072-6694/12/12/3574/s1, Table S1: Patient and tumor characteristics for NSCLC cohort, Table S2: List of the metabolites assigned and analyzed in serum samples of NSCLC patients, Figure S1: Bar plot reporting the values of Log2 (Fold Change, FC) of quantified metabolites. Metabolites with Log2 (FC) positive values have higher concentration in plasma samples from responder patients with respect to non-responders. Metabolites with Log2 (FC) negative values have lower concentration in plasma samples from responder patients with respect to non-responders. Red bars represent *p*-values < 0.05.
