# Supplementary material for: Metabolomics to Assess Response to Immune Checkpoint Inhibitors in Patients with Non-Small-Cell Lung Cancer
Source: Cancers (Basel). 2020 Nov 30;12(12):3574. doi: 10.3390/cancers12123574 (PMC7760033; doi:10.3390/cancers12123574)
Supplement: Supplementary file 1 [file cancers-12-03574-s001.pdf]

Supplementary Materials: Metabolomics to Assess Response to Immune Checkpoint Inhibitors in Patients with Non-Small-Cell Lung Cancer

Veronica Ghini, Letizia Laera, Beatrice Fantechi, Francesca del Monte, Matteo Benelli, Amelia McCartney, Tenori Leonardo, Claudio Luchinat and Daniele Pozzessere

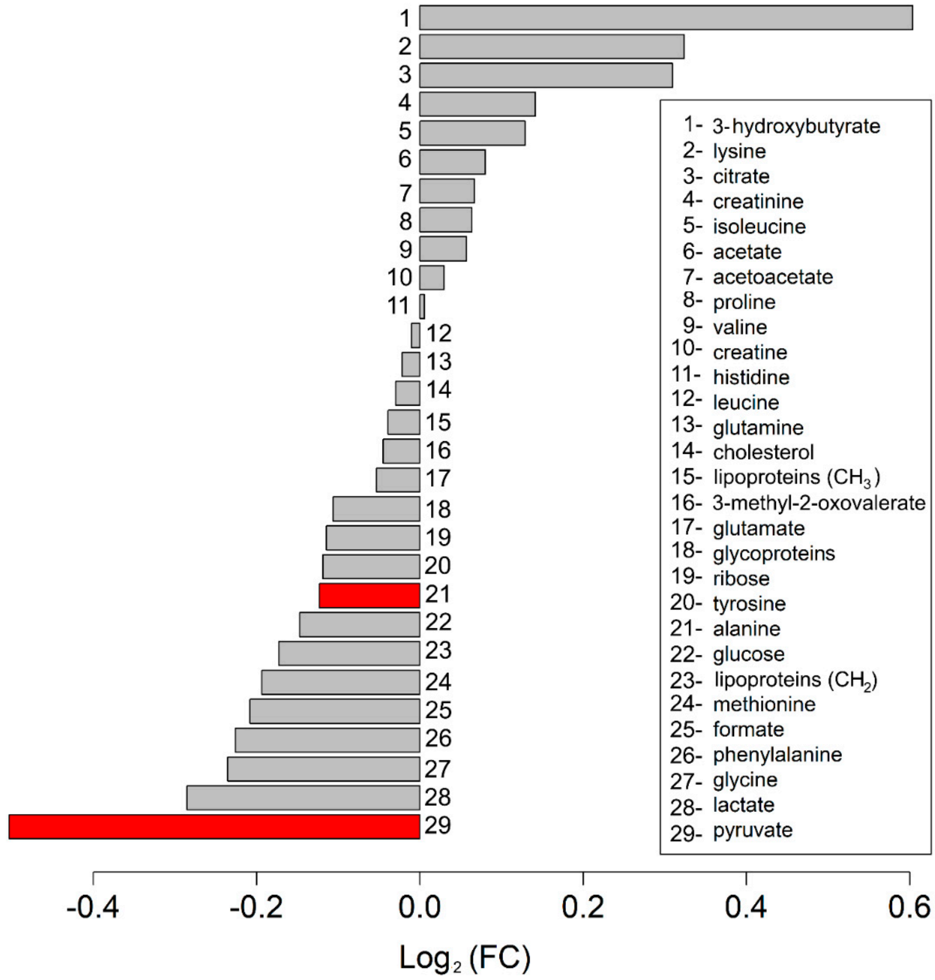

Figure S1. Bar plot reporting the values of Log2 (Fold Change, FC) of quantified metabolites. Metabolites with Log_2 (FC) positive values have higher concentration in plasma samples from responder patients with respect to non-responders. Metabolites with Log2 (FC) negative values have lower concentration in plasma samples from responder patients with respect to non-responders. Red bars represent p-values < 0.05.

Table S1. Patient and tumor characteristics for NSCLC cohort. NOS: not otherwise specified; SqCC: squamous histology; adeno: adenocarcinoma.

ID	Gender	Age	Histology	Site of Metastasis	Smoking Status	Drug
MetL01	F	60	NOS	lung, lymph nodes, adrenal, bone	Former	Niv
MetL05	M	69	SqCC	lung, trachea	Former	Niv
MetL06	M	69	SqCC	adrenal, colon	Current	Niv
MetL07	M	56	SqCC	lung, lymph nodes	Former	Niv
MetL09	M	45	SqCC	lung, bono, kidney	Current	Niv
MetL10	M	71	SqCC	lung, pleura	Former	Niv
MetL11	M	68	adeno	lung, lymph nodes	Current	Niv
MetL12	M	57	SqCC	lung, lymph nodes, bone, liver	Current	Niv
MetL13	F	75	adeno	lung, lymph nodes	Former	Niv
MetL15	F	73	adeno	lung, pleura	NA	Niv
MetL17	F	77	SqCC	lung	Former	Niv
MetL19	M	67	adeno	lung	Former	Niv
MetL20	F	74	SqCC	lung, lymph nodes, liver	Former	Niv
MetL22	M	59	adeno	lung, lymph nodes, bone, pleura	Current	Pem
MetL23	F	51	adeno	lung, lymph nodes	Current	Pem
MetL24	M	69	adeno	lung, bone	Former	Niv
MetL25	M	62	adeno	lung, lymph nodes	Former	Pem
MetL26	F	63	adeno	lymph nodes, bone	Former	Niv
MetL27	F	74	adeno	lung, lymph nodes, adrenal, bone	Never	Pem
MetL28	M	76	adeno	lymph nodes	Current	Pem
MetL29	F	70	SqCC	lung, pleura, bone	Current	Niv
MetL30	F	59	SqCC	lung, lymph nodes	Former	Niv
MetL31	M	84	adeno	lymph nodes, pleura	Former	Pem
MetL32	F	70	SqCC	lung, lymph nodes, adrenal, brain	Former	Niv
MetL33	M	77	SqCC	lung, lymph nodes	Former	Niv
MetL35	F	69	adeno	lung, lymph nodes, brain	Former	Pem
MetL34	F	48	adeno	lung, pleura, lymph nodes	NA	Niv
MetL36	M	76	adeno	lung, ureter, thoracic wall	Former	Niv
MetL37	M	75	SqCC	lung, lymph nodes, adrenal	Former	Niv
MetL38	F	81	adeno	lung, lymph nodes	Former	Pem

MetL39	M	68	adeno	lung, lymph nodes	Former	Niv
MetL40	M	71	adeno	lymph nodes, lung	Former	Niv
MetL41	M	73	SqCC	lung, lymph nodes, pleura, adrenal	Former	Niv
MetL42	F	70	SqCC	lung, lymph nodes	Current	Niv
MetL43	F	54	adeno	lung, lymph nodes, pleura, adrenal, bone	Current	Pem
MetL44	F	62	adeno	lung, lymph nodes, liver	Current	Niv
MetL45	F	71	SqCC	lung, lymph nodes, pleura, thoracic wall	Former	Niv
MetL46	M	61	adeno	lung, lymph nodes, brain	Current	Pem
MetL47	M	50	adeno	lung, lymph nodes, brain	Current	Pem
MetL48	M	68	SqCC	lung, lymph nodes	Former	Niv
MetL49	M	69	NOS	lung, liver, lymph nodes	Former	Niv
MetL50	M	83	SqCC	lung, lymph nodes	Former	Niv
MetL51	F	61	adeno	lung, brain	Former	Pem
MetL52	F	55	adeno	lung, lymph nodes, brain, adrenal	Former	Niv
MetL53	M	70	SqCC	lung, brain, liver, lymph nodes	Former	Niv
MetL55	M	72	adeno	lung, pleura, lymph nodes, bone	Current	Pem
MetL56	F	69	adeno	lung, brain, lymph nodes	Former	Pem
MetL57	M	56	adeno	lung, bone	Current	Niv
MetL59	F	37	adeno	lung, pleura	Never	Pem
MetL60	M	75	adeno	lung, bone	Current	Pem
MetL63	M	57	NOS	lung, brain, lymph nodes, adrenal	Current	Pem
MetL65	M	72	adeno	lung, lymph nodes	Current	Pem
MetL66	F	68	adeno	lung, pleura	Never	Pem

Table S2. List of the metabolites assigned and analyzed in serum samples of NSCLC patients. The Human Metabolome Database (HMDB) compound ID of each metabolite is reported.

Metabolites	HMDB ID
3-hydroxybutyrate	HMDB00011
3-methyl-2-oxovalerate	HMDB00491
Acetate	HMDB00042
Acetoacectate	HMDB00060
L-alanine	HMDB00161
Citrate	HMDB00094
Creatine	HMDB00064
Creatinine	HMDB00562
Cholesterol (CH3)	HMDB00067
Formate	HMDB00142
L-glycine	HMDB00123
D-glucose	HMDB00122
L-glutammate	HMDB00148
L-glutammine	HMDB00641
Glycoproteins (N-Acetyl)	-
L-histidine	HMDB00177
L-isoleucine	HMDB00172
Lactate	HMDB00190
L-leucine	HMDB00687
Lipoproteins (CH2)	-
Lipoproteins (CH3)	-
L-lysine	HMDB00182
L-methionine	HMDB00696
L-phenylalanine	HMDB00159
Pyruvate	HMDB00243
L-proline	HMDB00162
D-ribose	HMDB00283
L-tyrosine	HMDB00158
L-valine	HMDB00883

Publisher's Note: MDPI stays neutral with regard to jurisdictional claims in published maps and institutional affiliations.

© 2020 by the authors. Licensee MDPI, Basel, Switzerland. This article is an open access article distributed under the terms and conditions of the Creative Commons Attribution (CC BY) license (http://creativecommons.org/licenses/by/4.0/).
